# Transcriptome analysis of a cnidarian – dinoflagellate mutualism reveals complex modulation of host gene expression

**DOI:** 10.1186/1471-2164-7-23

**Published:** 2006-02-10

**Authors:** Mauricio Rodriguez-Lanetty, Wendy S Phillips, Virginia M Weis

**Affiliations:** 1 Department of Zoology, Oregon State University, Corvallis, OR 97331, USA; 2 Centre for Marine Studies, University of Queensland, St. Lucia, QLD 4072, Australia

## Abstract

**Background:**

Cnidarian – dinoflagellate intracellular symbioses are one of the most important mutualisms in the marine environment. They form the trophic and structural foundation of coral reef ecosystems, and have played a key role in the evolutionary radiation and biodiversity of cnidarian species. Despite the prevalence of these symbioses, we still know very little about the molecular modulators that initiate, regulate, and maintain the interaction between these two different biological entities. In this study, we conducted a comparative host anemone transcriptome analysis using a cDNA microarray platform to identify genes involved in cnidarian – algal symbiosis.

**Results:**

We detected statistically significant differences in host gene expression profiles between sea anemones (*Anthopleura elegantissima*) in a symbiotic and non-symbiotic state. The group of genes, whose expression is altered, is diverse, suggesting that the molecular regulation of the symbiosis is governed by changes in multiple cellular processes. In the context of cnidarian – dinoflagellate symbioses, we discuss pivotal host gene expression changes involved in lipid metabolism, cell adhesion, cell proliferation, apoptosis, and oxidative stress.

**Conclusion:**

Our data do not support the existence of symbiosis-specific genes involved in controlling and regulating the symbiosis. Instead, it appears that the symbiosis is maintained by altering expression of existing genes involved in vital cellular processes. Specifically, the finding of key genes involved in cell cycle progression and apoptosis have led us to hypothesize that a suppression of apoptosis, together with a deregulation of the host cell cycle, create a platform that might be necessary for symbiont and/or symbiont-containing host cell survival. This first comprehensive molecular examination of the cnidarian – dinoflagellate associations provides critical insights into the maintenance and regulation of the symbiosis.

## Background

Mutualistic symbioses are defined as the association between unrelated organisms living together in a close, protracted relationship that benefits both partners. They are common in nature and are a driving force in evolution [1]. Cnidarian – dinoflagellate associations represent one of the most important symbioses in the marine environment. These partnerships form the trophic and structural foundation of coral reef ecosystems, and have played a key role in the evolutionary radiation and biodiversity of cnidarian species.

The cnidarian host harbors algal endosymbionts, usually from the genus *Symbiodinium*, within gastrodermal cells in vacuoles of phagosomal origin known as the symbiosome. The initial infection occurs when host gastrodermal cells lining the gastric cavity phagocitize algal symbionts previously ingested through the host mouth during feeding [2]. The mechanisms of avoiding host digestion remain largely unknown, although some studies suggest that persistence may be due to the failure of phagosome-lysosome fusion [3-6].

This intracellular association is centered around nutrient exchange and is essential for both partners to thrive in tropical seas, an environment particularly low in nutrients. The algal endosymbionts can translocate up to 95% of their photosynthetic products to the hosts, where these compounds are primarily used to meet host respiratory demand [7,8]. In return, the symbionts receive protection from predation, nitrogen-based nutrients released from the host [8], and inorganic carbon for photosynthesis [9,10].

Despite the prevalence of these marine symbioses and the overall interest in coral reef health, we still know very little about the cellular and molecular basis of the intracellular cnidarian – dinoflagellate symbiosis. What are the key molecular modulators that initiate, regulate, and maintain the interaction between these two different biological entities? To date, only a few studies have examined either broad-scale patterns of RNA or protein expression in symbiotic cnidarians or identified specific genes that play a role in interpartner communication and regulation. Two dimensional proteomic analysis comparing symbiotic and naturally occurring symbiont-free (aposymbiotic) individuals of the Pacific coast temperate anemone *Anthopleura elegantissima *revealed dozens of proteins that were up- or down-regulated in the symbiotic state [11]. In subsequent studies, one strongly expressed symbiotic protein, sym32, was identified as belonging to the Fasciclin I protein family some of which function in cell-cell interactions or cell adhesion in other organisms [12]. In addition, immunocytochemistry and immunoblot studies using an anti-sym32 antibody found a putative homolog in the symbionts, leading to the suggestion that host-symbiont heterophilic fasciclin I interactions could be a method of interpartner signaling in the symbiosis [13]. Finally, in a recent study, a lectin has been identified and characterized from a symbiotic soft coral that may play a role in lectin-glycan signaling during onset of symbiosis [14].

**Figure 1:**
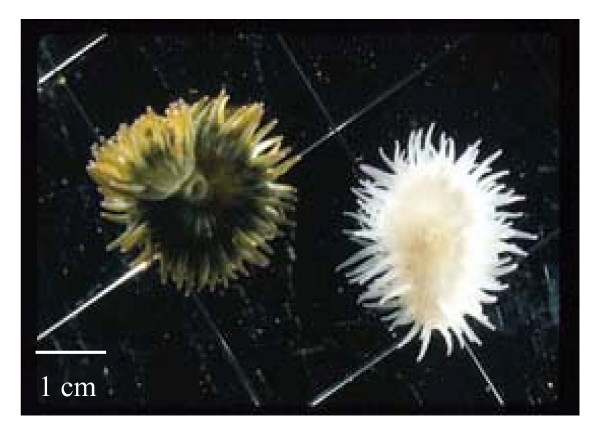
Photograph of a symbiotic (left) and aposymbiotic (right) *Anthopleura elegantissima*.

The discovery and identification of host genes that modulate cnidarian – dinoflagellate symbioses is a topic that is ideally suited to a comprehensive microarray approach. Recently, similar approaches have been used successfully to identify "symbiosis-specific" genes in other mutualistic associations such as rhizobial [15] and arbuscular mycorrhizal [16] symbioses. In this study, we conducted a comparative host anemone transcriptome analysis using a cDNA microarray platform to identify genes involved in cnidarian – algal symbiosis. Following earlier proteomic studies, we used the temperate anemone *Anthopleura elegantissima *as a model as it occurs naturally in both the symbiotic and aposymbiotic state (Figure 1). This first comprehensive molecular examination of the cnidarian – dinoflagellate association provides critical insights into the maintenance and regulation of the symbiosis.

## Results and discussion

**Figure 2:**
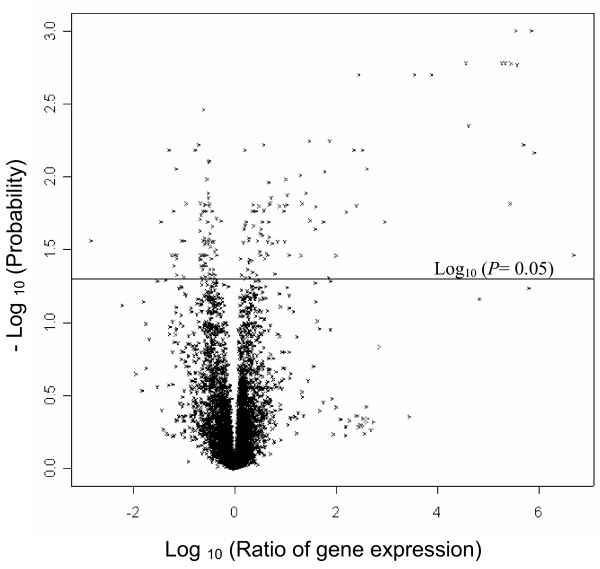
**Volcano plot displaying the probability and ratio of gene expression of all the 10,368 features on the array. **The spots above the probability threshold p < 0.05 (horizontal solid line) represent the 189 significantly different features. For better visualization, the log probability axis is shown with positive numbers

A total of 583 (5.62%) of the 10,368 features from the cDNA microarray were identified as significantly different between aposymbiotic and symbiotic anemones (*P *< 0.01). However, since 104 false positives were expected from the 10,368 gene-tests performed under the null hypothesis when *P *= 0.01, we applied a False Discovery Rate (FDR) multiple-testing adjustment to control for type I error [17]. Allowing for a 5% type I error for the whole set of significant genes, only 189 features, or 1.82% of the original 10,368 features, showed significant differences in expression between the aposymbiotic and symbiotic anemones (*P *< 0.05; Figure 2). After DNA sequencing and sequence analyses, these 189 features resolved into 91 unigenes.

**Figure 3:**
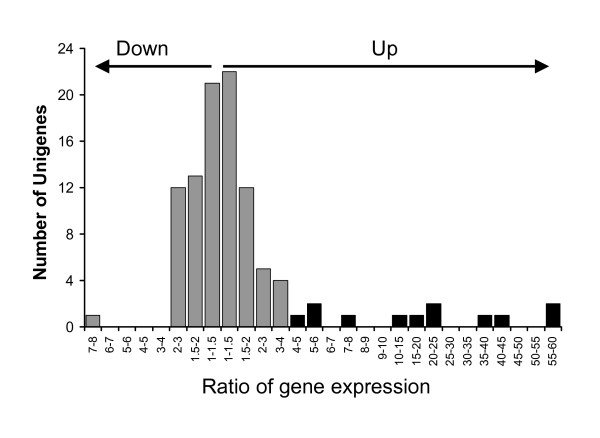
**Distribution of unigenes as a function of the ratio of expression between symbiotic and aposymbiotic anemones. **Arrows divide those down- and up-regulated in symbiosis. Black bars denote unigenes confirmed by PCR to be contaminating algal unigenes (see text for details).

Twelve of the 91 differential unigenes were very highly expressed in symbiotic state (Figure 3). These were viewed with suspicion as possible algal genes that were contaminating the host-only cDNA library. Specific primers for these highly symbiotic unigenes were constructed and used in PCR reactions with host-only and algae-only genomic DNA. Successful DNA amplification was achieved only in the algal DNA samples and not in the host genomic DNA (data not shown). These contaminating algal unigenes were therefore removed from further analyses.

The remaining 79 host unigenes showed differences in expression mostly ranging between 1 and 2 fold change. We hypothesize that these subtle changes in expression could be due in part to the use of whole animal mRNA extractions for the screening of the arrays. As the symbionts reside solely in the gastrodermal tissue of the host, a larger fold change of expression in the tissues which harbor symbionts could be hidden by non-differential expression of the same genes in non-symbiotic tissue. However, if genes were turned on or off in the whole animal as a function of symbiosis, we still would expect to see large changes in expression from the array data. We did not see such large fold changes of any genes. Future experiments comparing expression only in the gastrodermal tissue from symbiotic and non-symbiotic animals might show greater differences in expression in the same genes we describe here.

From our 79 identified host unigenes, only 28 (35%) showed significant BLAST hits (E < 1.0 × 10^-4^) with homologs to known genes in the Genbank (Table 1). Twenty three of these 28 genes matched more strongly to vertebrate homologs than to invertebrate homologs. This result is consistent with previous findings suggesting that cnidarian genomes contain many genes previously considered to be vertebrate innovations because of their absence from the *Drosophila *or *Caenorhabditis *genomes [18]. This provides further evidence for the unexpected paradox of genome evolution pointed out by Kusserow et al. [19]: "the gene diversity in the genomes of simple metazoans is much higher than previously predicted and some derived lineages such as flies and nematodes have a lower gene family diversity than simple metazoans."

Fifty one (65%) of our identified unigenes are unknown. This is consistent with other EST projects of other cnidarians that show a high number of unknown genes; 30% in the scleractinian coral *Acropora millepora *[18] and ~44% in the anemone *Aiptasia pulchella *[20]

**Figure 4:**
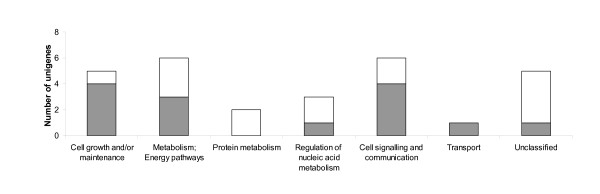
**Distribution of the known host unigenes based on their functional classification. **White and grey bars are the genes up- and down-regulated in symbiosis, respectively.

Rather than finding "symbiosis' genes which change expression as a function of symbiosis in an ON/OFF manner, we detected instead alterations in expression of genes regulating a broad array of functional processes (Figure 4, Table 1). Functional gene classification of the 28 differentially expressed known genes reveals the complex effect of symbiotic state on host gene expression. This suggests that symbiosis is regulated and controlled by changes within existing pathways used to control metabolism and growth of the animal as a whole rather than by pathways unique to the symbiotic state. Interestingly, despite the differences among mutualistic symbioses in nature, the molecular modulation of existing pathways in the host cell seems to be a key common factor in symbiosis regulation [21-23]. The functional interpretation of the 28 differentially expressed genes based on their functional classification led us to examine the pivotal expression changes involved in: 1) lipid metabolism, 2) cell proliferation and apoptosis, and 3) oxidative stress in the cnidarian – *Symbiodinium *symbiosis.

### Alterations in lipid metabolism

The mutualistic interaction between cnidarians and *Symbiodinium *has a nutritional basis involving the sustained and substantial bidirectional translocation of nutrients between the algal and animal host cells. It is well known that algae can release much of their photosynthetic carbon (>90%) to the animal host [7]. These photosynthates include carbohydrates, in the form of glucose [24], glycerol [25] and saturated and poly-unsaturated fatty acids [26,27]. In this study, expression changes of enzymes involved in host lipid metabolism provide indirect evidence suggesting that symbiotic anemones are indeed processing more lipids than are aposymbiotic anemones.

Phytanoyl-CoA hydroxylase, an enzyme involved in lipid degradation, which catalyzes the conversion of phytanoyl-CoA to 2-hydroxyphytanoyl-CoA in beta-methylated fatty acid metabolism [28], was more highly expressed in symbiotic compared to aposymbiotic anemones (1.54 fold from array data and 1.59 fold from Q-RT-PCR data). In contrast, the enzyme medium-chain S-acyl fatty acid synthase, involved in lipid synthesis, was down-regulated in symbiosis (1.56^-1 ^fold from array data and 1.32^-1 ^fold from Q-RT-PCR). Similar up-regulation of degradative enzymes and down-regulation of lipid synthesis enzymes, particularly the fatty acid oxidizing enzymes, have been detected in mammals in response to exogenous supply of fatty acids [29]. In the context of cnidarian – dinoflagellates symbioses, it makes sense that the host would not need to synthesize certain lipids if they are supplied from the symbiont, and that the host would turn on degradative machinery to break down the supplied lipids. In addition, we detected an up-regulation of the long chain acyl-CoA thioester hydrolase enzyme (1.28 fold from array data), the activity of which is also normally increased in mammalian animals when diets rich in polyunsaturated fatty acids are supplied [30]. This enzyme has also been implicated as an important signaling molecule in the regulatory cascade leading to fatty acid-mediated alterations in gene expression [29]. Further functional studies on cnidarian fatty acyl-CoA thioesters could shed light on the role of this compound in regulation of symbiosis.

The scavenger receptor class B type I (SR-BI, a member of the CD36 superfamily) is another differentially expressed gene also implicated in lipid metabolism. We detected an up-regulation of this scavenger receptor in symbiotic state (1.34 fold array data). SR-BIs are integral membrane glycoproteins found on the surface of a variety of cells that have functions ranging from fatty acid translocation to cell adhesion. We will discuss how the different functions described in other biological systems might also play a role in cnidarian – algal symbioses.

SR-BIs function in selective uptake and transport of cholesterol and other fatty acids from lipoproteins [31]. In the context of cnidarian – dinoflagellate symbioses, a cnidarian SR-BI homolog could facilitate traffic of the symbiont-derived fatty acid within and between host cells. Such a role is consistent with the increased expression of this protein in the symbiotic state, and with evidence that lipids are translocated from the algal symbiont to the host cell [27].

SR-BI (CD36) proteins are also involved in pathogen/parasite infection and cell adhesion. Recent evidence suggest that some pathogens such as *Mycobacterium *[32] and Hepatitis C virus (HCV) [33,34] take advantage of host SR-BI proteins and use them during the invasion of host cells. More specifically, Voisset et al. [34] showed a role in lipid transfer during HCV entry, which involves a complex interplay between SR-BI, high density lipoprotein and HCV envelop glycoproteins. Further, host SR-BIs facilitate adhesion of *Plasmodium*-infected human erythrocytes to uninfected erythrocytes, allowing for movement of the parasite between host cells [35]. A cnidarian SR-BI homologs could perform a similar function in facilitating dinoflagellate symbiont infection and transport from infected to uninfected host cells. This function is particularly intriguing with regard to cnidarian – dinoflagellates symbioses as *Plasmodium *is a member of the apicomplexa, a sister taxon to the dinoflagellates [36,37]. Future studies of immuno-fluorescence microscopy and immuno-inhibition of the cnidarian SR-BI homolog would help to localize and describe the function of this protein in cnidarian – algal symbiosis.

### Cell cycle regulation and suppression of apoptosis

The difference in expression between symbiotic and aposymbiotic animals of a key sphingolipid regulator suggests that symbiotic state contributes to suppression of apoptosis and expedition of host cell cycle progression. Transcripts of the enzyme sphingosine-1-phosphate phosphatase (SPPase), which is involved in the regulation of the sphingosine – sphingosine-1-phosphate (S1P) rheostat, were significantly down-regulated in symbiotic anemones (1.5^-1 ^fold from array data and 1.62^-1 ^fold from Q-RT-PCR data). The sphingosine – S1P rheostat determines whether a cell survives and proliferates or undergoes apoptosis and dies. It is regulated by two enzymes, sphingosine kinase which converts sphingosine to S1P and SPPase which catalyzes the opposite reaction.

**Figure 5:**
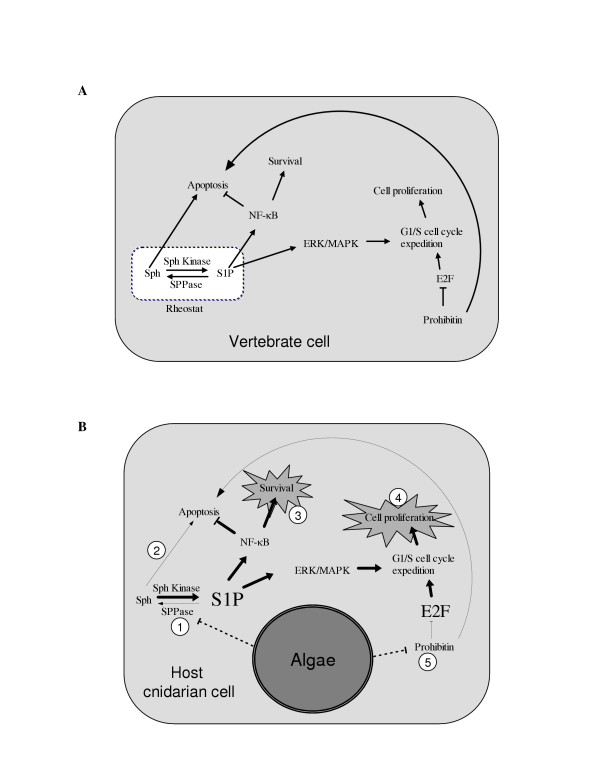
**Cellular regulation of Sphingosine-1-Phosphate (S1P) and Prohibitin. A**. Sphingosine (Sph) – S1P rheostat; showing the two enzymes that control it, sphingosine kinase and S1P phosphatase (SPPase); and regulation pathways of S1P and prohibitin in vertebrate cells. **B**. Model of S1P and prohibitin roles in cnidarian – algal symbiosis: (1) SPPase is down regulated by an unknown algal-induced mechanism, causing an increase of the levels of S1P over sphingosine; (2) the lower levels of sphingosine produce a suppression of sphingolipid-induced apoptosis; (3) higher levels of S1P enhance the activation of NF-κB, which inhibits apoptosis and increases cell survival; (4) S1P activates the ERK/MAPK pathway, which causes an expedition of G1/S cell cycle that results in cell proliferation; (5) Prohibitin is down-regulated by an unknown algal-induced mechanism, causing a suppression of apoptosis and a release of the blockage of E2F transcription factors. The latter enhances the expedition of G1/S cell cycle resulting in cell proliferation.

The sphingosine – S1P rheostat plays multiple signaling roles in higher animals (Figure 5a) [reviewed in [38]]. Increased levels of anti-apoptotic S1P enhance cell proliferation by expediting the G1/S transition in the cell cycle and by increasing DNA synthesis through the activation of transcription factors via extracellular-regulated kinase/mitogen-activated protein kinase (ERK/MAPK) pathways [39]. S1P also promotes the activation of the transcription factor nuclear factor κB (NF-κB), which plays a role in preventing apoptosis and enhancing cell survival [40]. Further, increased levels of S1P suppress apoptosis induced by high levels of pro-apoptotic sphingosine [38,39]

Based on the functions of the sphingosine – S1P rheostat in other organisms we have developed a model for its role in cnidarian – dinoflagellate symbioses (Figure 5b). We hypothesize that, by an unknown mechanism, the symbiotic state causes a down-regulation of host cell SPPase, thereby favoring the accumulation of S1P and reducing levels of sphingosine. High levels of S1P would in turn start a signaling cascade resulting in survival and proliferation of the host cell containing algae. Correspondingly low levels of sphingosine would result in a failure to initiate the apoptosis cascade, thereby promoting host cell survival.

Our model proposes regulation of the sphingosine – S1P rheostat by controlling levels of SPPase. If true, this would be only the second piece of evidence suggesting that the rheostat can be controlled via SPPase levels. Just one other study has demonstrated accumulation of S1P in mammalian cells by gene knockdown of SPPase [41]. All other studies of rheostat control have focused on changes in sphingosine kinase activity [42]. However, it is possible that the sphingosine kinase transcripts were not present in our anonymous cDNA array, and because of this we can not discard the possibility that this enzyme might also play a role in the modulation of the rheostat as a function of symbiosis.

The hypothesis that host/symbiont interactions are controlled in part by manipulation of host cell survival has been proposed in many pathogenic and parasitic interactions in animals [reviewed in [43]] and plants [44]. Of particular interest to our study is evidence that the tomato pathogen *Pseudomonas syringae *up-regulates the expression of a host cell signaling sphingolipid [45]. This in turn suppresses programmed cell death of the infected host cell thereby blocking an antimicrobial defense strategy used by many plant hosts.

In addition to observing symbiosis-specific differences in SPPase levels, we also observed a 2.2-fold down-regulation in the symbiotic state of prohibitin, another gene involved in the regulation of the cell cycle and apoptotic pathways (Figure 5a). Prohibitin is a highly conserved protein that inhibits cell proliferation and is a potential tumor repressor [46,47]. It is thought to act by repressing the E2F family of transcription factors, which promote the G1/S transition [48]. Furthermore, increases in prohibitin levels have been correlated with the initial events of apoptosis, however its molecular mechanism of action remains unclear [49]. Decreased expression of prohibitin in the symbiotic state fits well into our model for control of host cell proliferation (Figure 5b). Decreased prohibitin would result in increased transition into S phase and inhibition of apoptosis, resulting in symbiotic host cell survival and proliferation.

### Oxidative stress

We detected a down-regulation of a copper/zinc superoxide dismutase (CuZnSOD) in the symbiotic state (1.58^-1 ^fold from array data and 1.89^-1 ^from Q-RT-PCR data). Superoxide dismutases function as antioxidants that break down harmful superoxide radicals. In symbiotic cnidarians, a sequence for CuZnSOD has been characterized [50], and two other classes, MnSOD and FeSOD, have been described electrophoretically [51]. Previous findings have measured SOD enzyme activities nearly two orders of magnitude greater in symbiotic compared to aposymbiotic *Anthopleura elegantissima *[52]. One possible explanation for this apparently contradictory result is that the other SOD classes are being expressed specifically in the symbiotic state to mitigate the hyperoxic environment normally encountered in symbiosis. This explanation is in agreement with findings that show a low activity of CuZnSOD in the symbiotic anemone *Anemonia viridis *but an increased activity of FeSOD and MnSOD isoforms [51]. However, it has not been fully demonstrated that these Fe/Mn SODs are indeed of host origin. An alternative explanation for our results is that the host in the symbiotic state gains antioxidant protection from its symbionts, causing a reduction in expression of its own SODs. This would explain the down-regulation of CuZn SOD in the symbiotic state. Future studies may explore the origin of other symbiosis-induced SOD classes.

## Conclusion

In summary, we demonstrate that the gene expression profile of the host sea anemone *Anthopleura elegantissima *changes in symbiosis with the dinoflagellate *Symbiodinium muscatinei*. The group of genes whose expression is altered is diverse, suggesting that the molecular regulation of the symbiosis is governed by changes in multiple cellular processes. Our data do not support the existence of symbiosis-specific genes involved in controlling and regulating the symbiosis. On the contrary, it appears that the maintenance of the symbiosis is modulated by the alteration of expression of existing genes involved in vital cellular processes.

We show evidence that the gene expression of key biomolecules involved in cell cycle progression and apoptosis are differentially modulated in symbiosis. These results lead us to hypothesize that a suppression of apoptosis together with a deregulation of the host cell cycle create a platform that might be necessary for symbiont and/or symbiont-containing host cell survival. These findings have changed our perception of the cellular interaction between cnidarians and symbiotic dinoflagellates. We have always defined the cnidarian – algal interaction as cooperative since the ecological outcome of the interaction is a mutualistic symbiosis. However, at the cellular level the interaction between host and symbiont appears to have components of a parasitic or pathogenic interaction. Mutualistic symbionts, like pathogens, must overcome the host innate immunity to enter, reside, and grow inside the host cell. But intriguingly, though the algae-induced changes in the host cell show some similarity to pathogen – host interactions, they do not lead to the development of disease. This may be a common phenomenon in symbiosis, as a comparable picture is emerging from insect – bacteria [22] and squid – bacteria [53] symbioses. Understanding the nature of the molecular regulation of cnidarian – algal symbiosis by comparison with host/pathogen and host/parasite associations, it will provide further insight into the evolution of symbioses.

## Methods

### Collection and maintenance of experimental organisms

Specimens of *Anthopleura elegantissima *(Brandt) were collected in Neptune Beach, Oregon. Naturally-occurring aposymbiotic animals were collected from the intertidal zone in the shaded overhangs underneath the boulders. Symbiotic animals were collected from adjacent sun-exposed rocks. To standardize the physical condition to which the anemones were acclimated, the animals were maintained in the laboratory for four weeks in tanks of running seawater at ambient temperature (~14°C) and a light intensity of 100 μmol quanta m^-2^s^-1 ^on a 12 h light/ 12 h dark cycle. Animals were fed brine shrimp once a week. After this 4-week period, animals were frozen in liquid N_2 _and stored at -80°C. Algal dinoflagellates harbored by the symbiotic anemones were genetically identified, as *Symbiodinium muscatinei *sensu LaJeunesse and Trench [54] by sequencing and analyzing algal ITS rDNA following Rodriguez-Lanetty and Hoegh-Guldberg's protocol [55].

### Construction of cDNA arrays

Host-only RNA from four field collected aposymbiotic (apo) and symbiotic (sym) anemones was used to create apo and sym cDNA libraries in the Lamda Zap II bacteriophage vector (Stratagene). Algal symbionts were extracted and removed from sym anemones before host RNA extraction using previously described methods [12]. The Bluescript phagemid was excised and the plasmid library was plated onto LB-agar plates. Individual colonies were picked and grown in LB overnight in 384 well plates. The cDNA inserts were PCR amplifed using M13 forward and reverse vector primers. The size of cDNA inserts varied between 0.4 and 2.5 Kb. The redundancy of the libraries was checked by sequencing random clones. Within 73 sym clones, the redundancy was 16.4%; within 80 apo clones, the redundancy was 18.8%; and within all 153 clones, the redundancy was 29.4%.

To avoid bias in the random sampling of cDNA genes from *Anthopleura elegantissima*, similar proportions of library clones from the two conditions to be compared (aposymbiotic and symbiotic) were arrayed. A total of 10,368 PCR amplified cDNAs buffered in 3× SSC and 1.5 M betaine were spotted on UltraGAPS™ coated slides (Corning) without duplication. After printing, the arrays were dried for 48 h in vacuum desiccators, and UV cross linked at 300 mJ. The slides were stored in desiccators until the hybridization process.

### Hybridization of arrays

For probe construction, total RNA was extracted from aposymbiotic and symbiotic anemones (which included both host and algal partners) using Trizol, and then mRNA was isolated using the MicroPoly(A) Pure kit (Ambion). To confirm that samples were either symbiotic or aposymbiotic and to test sample quality, an initial cDNA synthesis was performed from the total RNA. That cDNA was used in quantitative RT-PCR reactions with anemone-specific actin primers and *Symbiodinium *rRNA primers. Symbiotic samples were used only if both actin and *Symbiodinium *rRNA amplified in high levels. In contrast, aposymbiotic samples were used only if actin was amplified in high levels and no amplication was detected of *Symbiodinium *rRNA.

cDNA probe synthesis was performed from 1 ug mRNA using Powerscript Reverse Transcripase (Clontech) and the Genisphere 3DNA-50 microarray kit according to the manufacturers' instructions. Slides for hybridization were chosen randomly from the batch of high quality printed arrays. Before hybridization, the slides were placed in isopropanol for 15 min, transferred to boiling water for 5 min, and dried via centrifugation. Apo and sym cDNAs were combined and hybridized to arrays in a formamide-based hybridization buffer under LifterSlips overnight at 50°C. Following post-hybridization washes, Cy3 and Cy5 Capture Reagents were hybridized to the array in the formamide-based buffer under LifterSlips for 3 h at 50°C. Following post-hybridization washes, slides were scanned using a GenePix ^® ^4200 scanner (Axon Instruments) and image acquisition and quality control was performed using the software GenePix ^® ^Pro 5.

### Experimental design and statistical analysis of microarray data

We applied a multiple dye-swap experimental design for the two conditions, aposymbiotic and symbiotic anemone groups, compared in our experiment [56]. Six biological replicates per condition were used as recommended for this type of two-comparison experimental design [17] (a total of 11 microarrays, [Gene Expression Omnibus: GSE3958]). Ratio-Intensity plots were constructed for each array data to explore whether or not intensity dependence of log ratios, which appears as curvature, was present. The assumption from cDNA microarray data is that most genes are not differentially expressed among treatments, and therefore most points in the RI plots should fall along a horizontal line centered on zero. Because curvatures were detected in a few of the arrays, an rLowess curve fitting transformation [57] was applied to the data. The transformation was applied to all the arrays to keep consistence in the whole data set as suggested by Cui et al. [58]. To detect differentially expressed genes between the two conditions, the following 2-stage ANOVA mixed model [17] was fitted to the log transformed intensity data; Y= A (Array) + D (Dye) + S (Sample = Individual) + C (Condition) + E (Error). In this mixed model, the arrays and samples were treated as random factors, and dye and condition as fixed factors. The Fs statistic, a shrinkage estimator for gene-specific variance components that makes no assumptions about the distribution of variances across genes, was estimated [59]. After the statistics were estimated, *p*-values were calculated from random permutations (N. permutations = 500 with sample reshuffling), and adjusted to correct for Type I error derived from multiple testing using the false discovery rate (FDR) approach [17]. All the analyses described above were conducted using the software R/Maanova [17].

### Sequencing and gene identity

Differentially expressed cDNA inserts were sequenced from both directions. Using the Seqmerge program from the Wisconsin Package Version 10.3 (Accelrys Inc., San Diego, CA), vector and low quality sequences were masked manually and sequence fragments were assembled into contigs. The contigs and their fragments were manually checked within Seqmerge to ensure an accurate consensus sequence. A blastx search of NCBI non-redundant proteins was performed for each contig, and those with expect values of less than 1 × 10^-4 ^were considered to be homologs to the matched known protein. For each gene, we report the hit with the lowest expect values that are to a known protein.

### Validation by quantitative real time PCR

Specific primers amplifying approximately 100–200 bp PCR products were designed for some of the genes as indicated later in the text, and specific amplification of appropriately sized bands was checked (Table 2). cDNAs from each of five aposymbiotic and symbiotic anemones were synthesized from 100 ng of mRNA and then diluted to a final volume of 330 μl. Two μl of cDNA was used in triplicate 20 μl quantitative RT-PCR reactions with 250 nM primers and iQ SYBR Green Supermix (BioRad) for a total of 40 cycles. The comparative delta CT method corrected for the actual PCR efficiency was used to determine relative quantities of mRNA transcripts from each sampled anemone. The PCR efficiency was determined using LinRegPCR [60]. For normalization purposes, we used multiple house-keeping genes and calculated a normalization factor from the geometric mean of their expression levels, as proposed by Vandesompele et al. [61]. To identify house-keeping gene controls, we selected 8 genes from our microarray platform that did not show significant differences between the treatments and whose ratios of expression were not significantly different from one (single t-test, α = 0.05). These putative house-keeping genes were then tested for their expression stability using geNorm [61]. The three most stable genes were used to calculate the normalization factor for each of the cDNA samples. These three house keeping genes are beta-actin [GenBank: DQ314617], a putative senescence-associated protein [GenBank: DQ314618], and ribosomal protein L12 mRNA [GenBank: DQ314616] (Table 2). Statistical analysis was conducted using a permutation t-test on the normalized data.

## Authors' contributions

MRL conceived the experimental design and carried out part of the molecular genetic study, performed the statistical analyses, and drafted the manuscript. WSP carried out most of the molecular genetic study, performed the sequence alignments and gene database blasts. VMW conceived the study, and participated in the design and coordination of the study. All the authors read and approved the final manuscript.

## Figures and Tables

**Table 1:** Known unigenes showing significant expression differences between symbiotic and aposymbiotic anemones. Identification was based on significant blast hits (E values < 10^-4^) from GenBank. Ratios of expression from all microarray analyses and selected Q-RT-PCR analyses are shown. Arrows indicate the direction of expression as a function of symbiosis: up- or down-regulation. Those genes highlighted in bold are discussed in the text. Significance is indicated as follows: *P *< 0.05 (*), *P *< 0.01 (**).

Unigenes grouped based on functional category	**Genbank Accession ID**	**OSU-CGRB ID**	**Expression as function of symbiosis**	
			
			Microarray	Q-RT-PCR
*Cell Growth and Maintenance*				
von Willebrand factor	DQ309533	AeOSU006130	↑ 1.92 *	↑ 1.82 *
Tubulin alpha chain	DQ309523	AeOSU007825	↓ 1.20 *	
Myosin regulatory light chain 2	DQ309534	AeOSU008456	↓ 1.37 *	
Beta-tubulin	DQ309552	AeOSU009795	↓ 1.39 *	
Collagen alpha-1 chain, type XII	DQ309546	AeOSU000073	↓ 1.94 **	

*Metabolism and Energy Pathways*				
**Phytanoyl-CoA hydroxylase**	DQ309528	AeOSU005830	↑ 1.54 *	↑1.59 *
Selenoprotein W1	DQ309535	AeOSU002895	↑ 1.42 *	
Long chain acyl-CoA thioesterase	DQ309538–DQ309539	AeOSU003383	↑ 1.28 *	
ATP synthase, H^+ ^transporting, mitochondrial F0 complex	DQ309536	AeOSU000538	↓ 1.29 *	
**Medium chain S-acyl fatty acid synthetase thio ester hydrolase**	DQ309537	AeOSU000658	↓ 1.56 *	↓ 1.32 *
**Copper/zinc superoxide dismutase (SOD)**	DQ309550	AeOSU001001	↓ 1.58 *	↓ 1.89 *

*Protein metabolism*				
60S ribosomal protein L26	DQ309540	AeOSU004867	↑ 3.43 **	
Plasma glutamate carboxypeptidase	DQ309521	AeOSU001186	↑ 1.70 *	

*Nucleic Acid Metabolism*				
Activating transcription factor 4 (ATF4)	DQ309526	AeOSU001247	↑ 1.51 *	↑ 2.49 *
Histone H3.3	DQ309522	AeOSU007640	↑ 1.22 *	
DEAD box polypeptide 48	DQ309551	AeOSU006260	↓ 1.30 *	

*Signal Transduction *&*cell communication*				
Oncoprotein-induced transcript 3	DQ309527	AeOSU009888	↑ 1.40 *	
**Scavenger receptor class B, type 1 (CD36)**	DQ309525	AeOSU008133	↑ 1.34 *	
**Sphingosine 1-phosphate phosphatase 2**	DQ309531–DQ309532	AeOSU005519	↓ 1.50 *	↓ 1.63 *
Betaine homocysteine methyl transferase	DQ309529	AeOSU002910	↓ 1.56 *	↓ 4.18 *
Calcium/calmodulin-dependent protein kinase IV	DQ309544	AeOSU005891	↓ 1.57 *	
**Prohibitin protein**	DQ309542–DQ309543	AeOSU001676	↓ 2.21 *	

*Transport*				
Membrane-bound transferrin-like protein p97	DQ309541	AeOSU001619	↓ 1.29 *	

*Unclassified*				
Death-associated protein 1 (DAP-1)	DQ309530	AeOSU008955	↑ 1.39 *	
Human headcase homolog (HECA)	DQ309548	AeOSU009119	↑ 1.33 *	
Guanine nucleotide binding protein, gamma 5 or 12	DQ309547	AeOSU008880	↑ 1.29 *	
SEC31-like 2 isoform A	DQ309524	AeOSU009211	↑ 1.17 *	
Methylmalonate semialdehyde dehydrogenase	DQ309545	AeOSU005938	↓ 1.37 *	

**Table 2:** Forward and reverse primers used to amplify the following genes in Q-RT-PCR assays. House keeping genes are indicated with (*).

Gene	Primer sequence
von Willebrand factor	For: CTG CCA AGA GGC GAA TAT GT Rev: GCA CAT GTT CTG GTC CCT CT
Phytanoyl-CoA hydroxylase	For: GCC AGC GCA CAC TGT TAT TA Rev: TTT CCT TGC ACA AGA CGT TG
Medium chain S-acyl fatty acid synthetase thio ester hydrolase	For: GAT AAA ACA CTG GGG AGG AAC Rev: CTT GTT ACT TGA CTC CAA CCT TC
Copper/zinc superoxide dismutase (SOD)	For: CTC AGG AGG GTG ATG GAA AA Rev: TTG CCG AAG GGA TTA AAG TG
Activating transcription factor 4 (ATF4)	For: GCG TCC AAG AGA GAA ACA CC Rev: CGA TAT CTT GTG GCA GCA TC
Sphingosine 1-phosphate phosphatase 2	For: TTG TGG CAC GCA CTC TTT AC Rev: TCC CAT TGG TAT CGC TTT TC
Betaine homocysteine methyl transferase	For: ACC AGG TGG GAT ATG CAA AA Rev: CCC CAC ATT TCA TGC TTT TC
Beta-actin *	For: CTG ATG GAC AGG TCA TCA CCA T Rev: CTC GTG GAT ACC AGC AGA TTC C
Putative senescence-associated protein *	For: TCG CAC CAT TGG CTT TTC Rev: CCT ATC ATT GCG AAG CAG AA
Ribosomal protein L12 mRNA *	For: CCT AAG TTC GAC CCC AAT GA Rev: TTT TGG ATG GTC AGT TGG AC
